# A clinician’s handbook for using ctDNA throughout the patient journey

**DOI:** 10.1186/s12943-022-01551-7

**Published:** 2022-03-21

**Authors:** Samantha O. Hasenleithner, Michael R. Speicher

**Affiliations:** 1 Institute of Human Genetics, Diagnostic and Research Center for Molecular BioMedicine, Medical University of Graz, Neue Stiftingtalstr. 6, 8010 Graz, Austria; 2 BioTechMed-Graz, Graz, Austria

**Keywords:** Cell-free DNA, Circulating tumor DNA, Liquid biopsy, Next-generation sequencing, Precision oncology, Whole-genome sequencing, Open chromatin

## Abstract

**Background:**

The promise of precision cancer medicine presently centers around the genomic sequence of a patient’s tumor being translated into timely, actionable information to inform clinical care. The analysis of cell-free DNA from liquid biopsy, which contains circulating tumor DNA (ctDNA) in patients with cancer, has proven to be amenable to various settings in oncology. However, open questions surrounding the clinical validity and utility of plasma-based analyses have hindered widespread clinical adoption.

**Main body:**

Owing to the rapid evolution of the field, studies supporting the use of ctDNA as a biomarker throughout a patient’s journey with cancer have accumulated in the last few years, warranting a review of the latest status for clinicians who may employ ctDNA in their precision oncology programs. In this work, we take a step back from the intricate coverage of detection approaches described extensively elsewhere and cover basic concepts around the practical implementation of next generation sequencing (NGS)-guided liquid biopsy. We compare relevant targeted and untargeted approaches to plasma DNA analysis, describe the latest evidence for clinical validity and utility, and highlight the value of genome-wide ctDNA analysis, particularly as it relates to early detection strategies and discovery applications harnessing the non-coding genome.

**Conclusions:**

The maturation of liquid biopsy for clinical application will require interdisciplinary efforts to address current challenges. However, patients and clinicians alike may greatly benefit in the future from its incorporation into routine oncology care.

## Background

Genomics currently serves as the backbone of the precision medicine construct. In cancer, systematic analyses of tumor genomes have allowed us to describe malignancies at the molecular level, in particular enabling the identification of driver events that propel disease. Novel therapies have been developed to treat these genomic driver events, which has led to improved patient outcomes across a spectrum of tumor types [1–4]. Given that tumors may evolve under the selective pressure of therapy, rich reservoirs of critical and real-time genomic information can be accessed through non-invasive means, i.e., liquid biopsies. Liquid biopsies rely upon detection of circulating tumor cells, cell-free DNA (cfDNA), which in patients with cancer includes circulating tumor DNA (ctDNA), RNA, proteins, lipids, and metabolites present in biofluids of patients. In principle, bodily fluids other than plasma, such as cerebrospinal fluid, urine, saliva, stool, pleural fluid, and ascites, can be analyzed, but for reasons of brevity, we focus here on blood and only on cfDNA. However, widespread clinical adoption has been slow, which is in part caused by an increasing complexity in selecting the most suitable analyses for the clinical question at hand, interpretation of the results, and lagging clinical trial evidence of utility. In this review, our intention is to especially target clinicians and we therefore cover concepts around practical implementation of next generation sequencing (NGS)-guided liquid biopsy while simultaneously highlighting the emerging new developments in the field. In this work, we break down ready-to-use ctDNA applications, including an overview of present clinical validity and clinical utility, describe early detection strategies, cover industry trends, and highlight exciting future directions and open questions that extend beyond DNA sequence.

## Main text

### A wealth of information circulating in the peripheral blood

Since the original description of abnormally high levels of circulating cell-free DNA (cfDNA) in the blood of cancer patients [5], further research has demonstrated that extracellular DNA in bodily fluids may reflect an array of pathological processes, including malignant, inflammatory or autoimmune disease, as well as trauma, sepsis and myocardial infarction [6–10], conditions which are outside the scope of this review. In the case of cancer, apoptotic or necrotic cancer cells release DNA into the bloodstream that can then be detected through diverse means, albeit always in the background of cfDNA molecules originating from the hematopoietic system, as these cells are the main contributors of DNA to the circulation in both health and disease [11, 12].

In addition to their eased access, liquid biopsies may capture the tumoral spatial heterogeneity not observed from traditional single-site biopsy genotyping [13–16], as they may enable the detection of DNA shed from both clonal and subclonal sites within multiple metastatic lesions. An array of studies has established the general concordance between aberrations detected in ctDNA and tumor tissue, ranging approximately between 70 and 90% [17–21]. Some discordance between mutations identified in primary tumor tissue and ctDNA is to be expected, which can be attributed to tumor heterogeneity or evolution, sampling bias, time lapses between sample acquisition, differences in sensitivity of the sequencing assays applied, or even different sequencing platforms [22–26]. However, with suitable and validated workflows, the potential applications of ctDNA are far-reaching, including diagnosing cancers earlier than traditional imaging [27–29], customizing treatments detected via genotyping [30–33], associating DNA levels with response to treatment [34, 35], identifying mechanisms of resistance to therapies [36–41] and measuring minimal residual disease after treatment [42–48]. As new evidence of analytical validity, clinical validity as well as utility continues to accumulate for these applications, strategies and requirements for the integration of ctDNA analysis workflows into clinical oncology programs are taking form [49–53].

### Next-generation sequencing of plasma DNA allows for diverse detection modalities of ctDNA

In order to better understand the novel ctDNA profiling strategies described herein later on, we briefly summarize basic concepts and assays used in NGS-based detection of ctDNA, as other diverse detection methodologies and their features have been reviewed in depth elsewhere [49, 50, 54–56].

#### Sampling, sequencing and detection of alterations from cfDNA

**Fig. 1:**
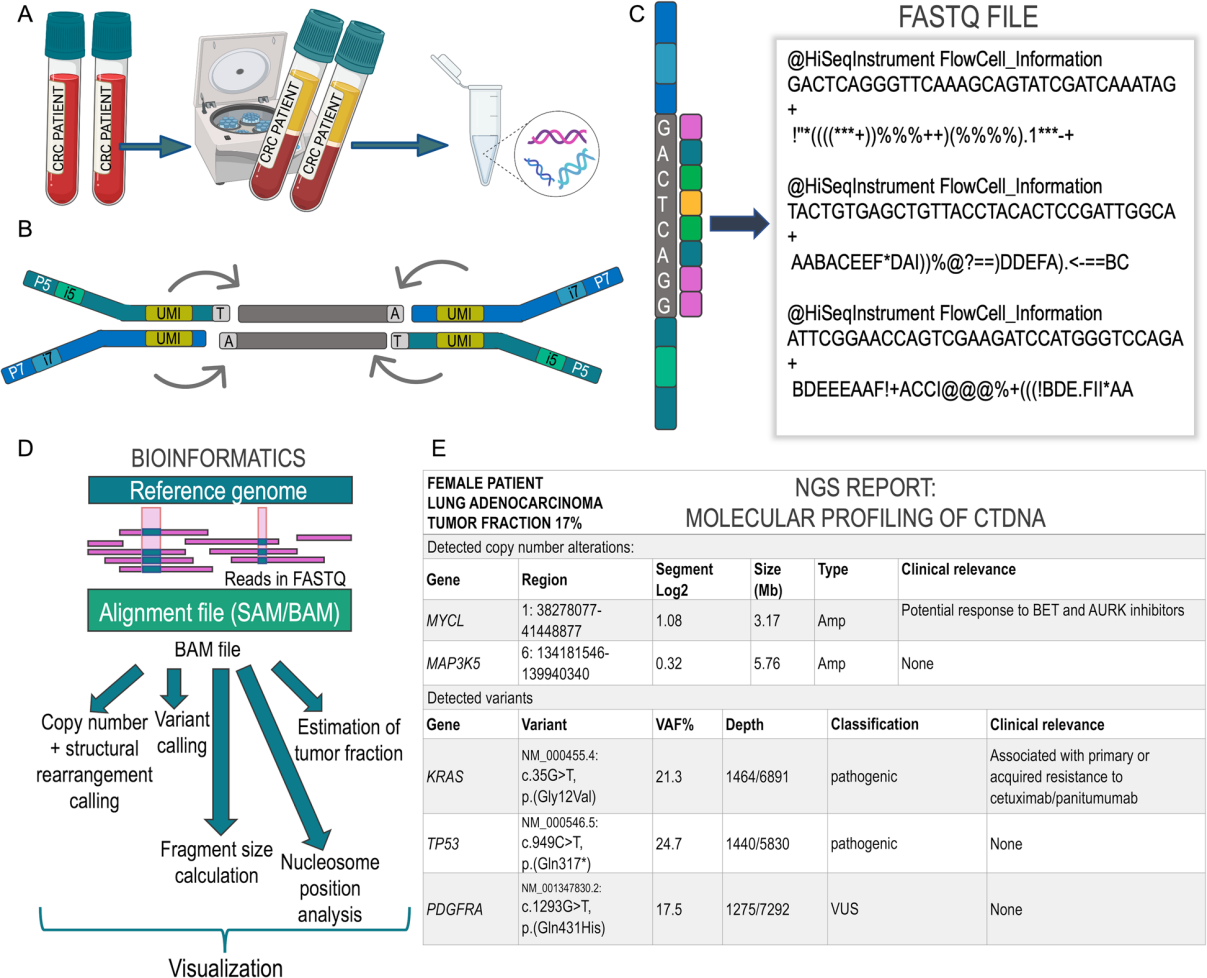
NGS technology as the backbone of ctDNA analysis. **A** Starting with whole blood collected in specialized cfDNA collection tubes, the plasma layer containing cfDNA is separated via centrifugation, followed by extraction of cfDNA from plasma. Typically, two vials of blood corresponding to ~17-20ml are submitted for analysis for both research studies or analysis by commercial vendors to ensure that sufficient amounts of plasma are available for extraction and harvesting of the ctDNA signal. **B** Simplified theoretical (Illumina) library fragment as a result of NGS library preparation. The dark green and dark blue bars represent the Illumina adapters P5 and P7, respectively, which enable hybridization to the sequencing flow cell and subsequent bridge amplification after ligation to the cfDNA fragment (gray bars). Sample-specific indexes, which are used to identify the patient sample, are typically in dual format and are shown here as i5 and i7. Additionally, unique molecular identifiers (UMI) serve as molecule-specific barcodes that enable the bioinformatics filtration of amplification or sequencing errors to ensure high-quality variant calling. **C** Sequencing-by-synthesis (SBS) on an Illumina instrument allows for one fluorescently-tagged nucleotide to be added to the growing read per cycle. Here, G’s, A’s, C’s and T’s are tagged with pink, blue, green and yellow fluorochromes, respectively. After the instrument has converted the captured images to base calls, the data is converted into a FASTQ file containing reads and quality scores. **D** Since the reads in the FASTQ file do not describe genomic location of the read, the data must first be aligned to a reference genome. This alignment is referred to as a SAM file, which has a binary counterpart called a BAM file. The BAM file contains all information in the original FASTQ file along with the mapping information of the read, i.e. the genomic coordinates to which it aligned. The BAM (alignment) file serves as the core data for diverse downstream analyses, e.g. calling of SCNAs or variants, estimation of tumor fraction from plasma, calculation of fragment size distributions, or nucleosome mapping. **E** Example of a clinical report summarizing the interpretation of genomic alterations detected from cfDNA. Such a clinical report describes the detected genomic alterations alongside their variant allele frequencies (VAF) and pathogenicity with potential clinical implications. Such findings should be discussed at a molecular tumor board and aligned to the patient’s clinical status

Sample acquisition begins with the collection of the peripheral blood, typically drawn in specialized collection tubes, e.g. PAXgene Blood ccfDNA tubes or those provided by a commercial provider, which contain an additive that stabilizes blood cells and prevents cell lysis. This aspect is critical, as any lysis of healthy blood cells will generate even more background signal that dilutes the probability of capturing the essential tumor-specific signal downstream [57–62]. After plasma separation, DNA extraction and quantification (Fig. 1 A), the selection of the approach to library preparation dictates what type of information can be harvested from the analysis. All NGS approaches, regardless of analyte, i.e., DNA from tissue or plasma DNA from whole blood, typically follow the same general workflow [63, 64]. The basic protocol begins when adapters of known sequence are added to the isolated DNA, which is then amplified into a library of DNA fragments (Fig. 1B). These adapters serve a technical rather than a biological purpose, as they enable the binding of amplified library fragments to the glass flow cell on which—in the case of Illumina instruments—sequencing-by-synthesis (SBS) takes place. The sequencer then converts the nucleotide-level biological information into a digital readout, which is stored in a large text file consisting of individual reads of pre-determined length (Fig. 1C). These reads, essentially a string of A’s, T’s, C’s and G’s, are then processed through various computational analysis pipelines to derive genomic variance from a reference genome (Fig. 1D). This variance must be interpreted within the context of the tumor and ctDNA fraction in plasma. For discussion at molecular tumor boards (MTBs), only the most pertinent information is summarized and prioritized in a clinical report such that actionability of alterations is displayed clearly for the expert panel (Fig. 1E) [65–68].

#### The impact of ctDNA levels, sequencing depth and breadth on ctDNA detection capabilities

Factors that critically affect the probability of ctDNA detection include the ctDNA levels in a plasma sample, the sequencing depth at mutant positions and the number of alterations tracked, i.e., the breadth of analysis.

The variable fractions of ctDNA among total cfDNA populations naturally have an effect on variant allele frequencies (VAFs). VAFs, reported as percentages, define the variant mutant reads over the total number of reads in a sample and are dependent not only on a patient’s tumor burden, but also on tumor fraction in plasma and tumor heterogeneity. However, a general problem is the low abundance of ctDNA fragments in many samples, particularly in early-stage disease. For this reason, detecting lower allelic frequencies—i.e. VAFs < 1% or even lower—is a basic requirement for cfDNA assays. Hence, highly sensitive approaches are needed to attain low detection limits for analyzing minute amounts within cfDNA. Importantly, the presence of ctDNA even at low levels, such as 0.1%, means that millions of actively dividing cancer cells are present within the body [69]. In contrast, in advanced disease, higher VAFs facilitate robust detection in most samples [24, 70].

Sequencing coverage, sometimes referred to as “depth”, describes the number of unique reads that align to, i.e., cover, nucleotide bases in a reference genome. After alignment of reads to the reference genome, we can observe how many of these reads support a particular locus. At higher levels of coverage, each base is covered by a greater number of aligned sequencing reads, therefore increasing the degree of confidence that base calls can be made at a given position or region. Importantly, deeper coverage improves the sensitivity of calling alterations and especially enhances the detection of rare variants that may be attributed to both tumor heterogeneity and/or low tumor fraction in a plasma sample.

The breadth refers to increasing the number of detectable sites, which can be achieved by increasing the number of regions in targeted panels or conducting whole-exome (WES) or whole-genome sequencing (WGS). The reasoning is that the detection of a single somatic mutation depends on the probability that the mutated fragment is actually sampled within the limited number of genome equivalents (GEs) present in a typical plasma sample [50]. In contrast, the probability to detect at least one somatic tumor-associated variant increases with the number of mutations analyzed so that the breadth of sequencing can compensate the limitation of low numbers of ctDNA fragments in plasma samples.

### Selecting the most appropriate assay

**Fig. 2:**
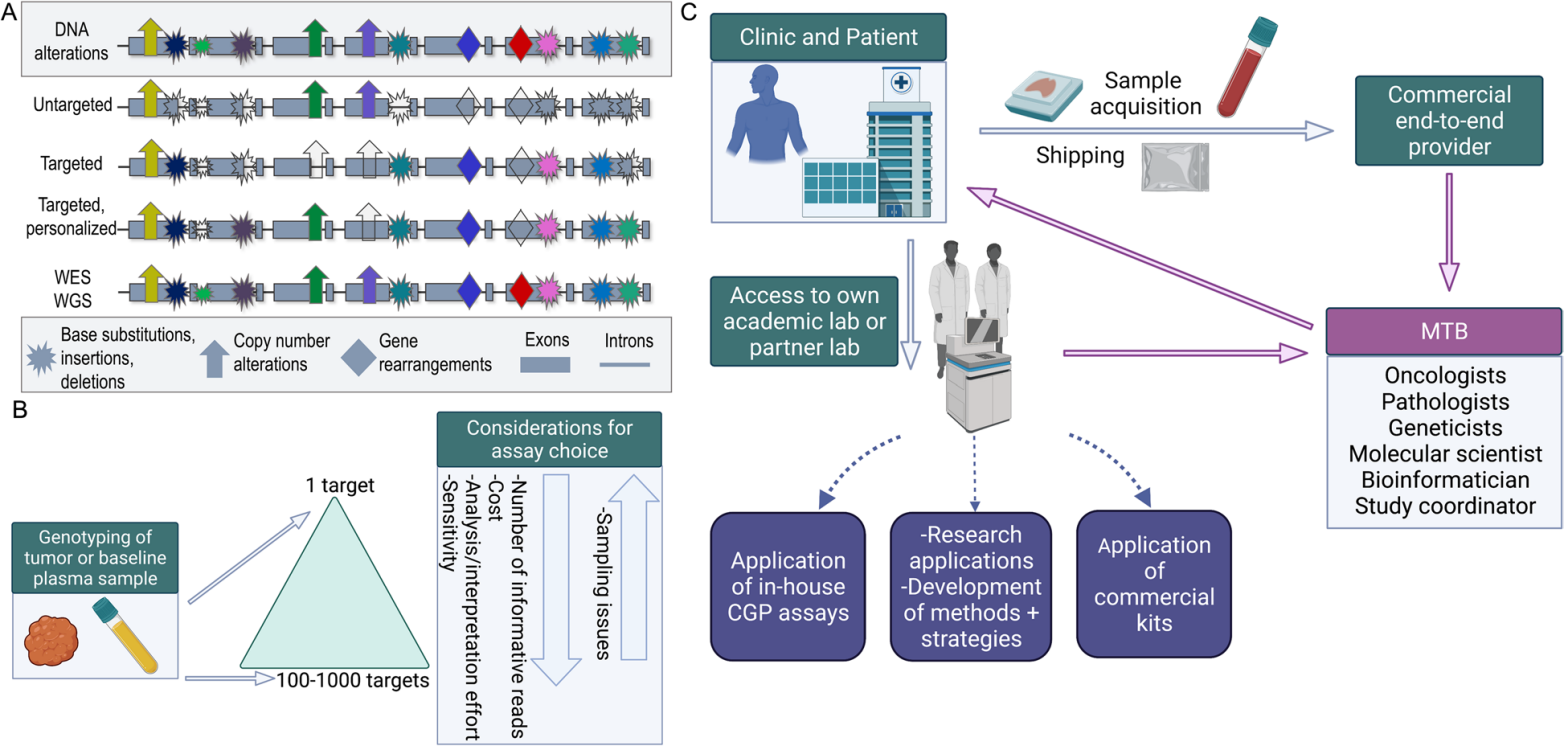
Choosing the right ctDNA assay based on sensitivity and breadth of genome coverage. **A** The number of detectable alterations critically depends on the selected cfDNA assay. The first row illustrates a DNA segment with various alterations (explained in the bottom legend). The second row (untargeted profiling) indicates the use of an “off the shelf” panel, which is capable of identifying a number of alterations, but as it represents a rather general assay, it may miss a considerable number of alterations (indicated by empty symbols). The third row (targeted profiling) indicates the use of a panel tailored for a specific tumor entity. For example, after screening of databases such as COSMIC and TCGA, panels can be designed that will identify specific alterations for this particular tumor entity with high likelihood. However, mutations “private”, i.e. unique, to the patient’s tumor will be missed. The fourth row (targeted, personalized profiling) indicates the use of a patient-specific multiplex assay, which was individually designed based on sequencing information from the primary tumor. In theory, all mutations from the primary tumor are detectable; however, new alterations that may have occurred at a later timepoint will be missed. The fifth row shows the use of whole-exome sequencing (WES) or whole-genome sequencing (WGS), which enables comprehensive coverage of all coding regions of the genome and, in case of WGS, also of all non-coding regions. The actual capability of detecting variants does not solely depend on the selected assay but also on other factors such as the ctDNA levels. **B** For a personalized approach, the tumor or a baseline plasma sample needs to be sequenced first. The observed mutations can then be leveraged for subsequent cfDNA analyses. The triangle in the center indicates the various breadth of such analyses. Advantages of analyzing only a single locus include low costs and easy interpretation without the necessity of sophisticated bioinformatics. However, sensitivity is limited, as sampling issues represent a significant confounding factor. In contrast, analyses of hundreds or thousands of targets requires some error-suppression means, i.e., bioinformatics tools. At the same time, the likelihood for the detection of evidence for the presence of ctDNA increases tremendously, making such approaches the most sensitive for MRD detection. In fact, while sequencing depth remains a critical factor for ctDNA detection, sequencing breadth may supplant the importance of high coverage analyses. **C** Some clinics may have access to their own academic or partner laboratory, which may develop and apply its own tests and address liquid biopsy related research questions. Alternatively, samples can be sent to a commercial end-to-end provider. Regardless which laboratory conducts the analyses, the aim is to provide the MTB with all relevant information at hand so that the best decisions can be made for patients

In general, ctDNA analyses have two key objectives. One is to detect evidence for the presence of ctDNA in the circulation with the highest possible specificity and sensitivity. The second is, in addition, a detailed characterization of the tumor genome, for example to identify druggable targets, resistance markers, or to follow the evolution of the tumor genome during a disease course. Key concepts in assay design (Fig. 2A) include first, whether an assay is targeted, i.e., focusing on particular regions in the genome according to specific criteria, or whether it is untargeted, which may be the case when WES or WGS is conducted. Second, assays can be personalized, i.e., tailored for an individual patient based on sequencing data obtained from the primary tumor or a baseline plasma sample (Fig. 2A,B), or they can be non-personalized, i.e., tumor-agnostic, which means that they are conducted without *a priori* knowledge of alterations. The selection of the assay depends on the objective and the associated costs plus turnaround time to obtain the results.

#### Targeted, non-personalized approaches: from single locus to panel applications

Single-locus PCR assays for known mutations have the potential to detect those with high sensitivity, in particular if analyzed with a several thousand-fold coverage (i.e., high depth, low breadth). For example, hotspot mutations in *KRAS* can easily be tracked in cases where *KRAS* is involved with high prevalence, such as in pancreatic or colon cancer. However, sampling issues may affect single-locus approaches, e.g., the region may be missed in samples that have low fractional concentrations of ctDNA, such that usually only a reliable detection limit of ~ 0.1% can be achieved. Hence, targeting a larger number of variants, i.e., increasing the breadth, has the potential to increase the sensitivity of ctDNA assays. For this reason, a number of panel sequencing assays capable of deeply sequencing a variable number of actionable mutations and genes have been developed.

Targeted sequencing approaches offer a cost-effective solution to interrogate only those loci of interest, such as clinically relevant hotspot mutations, that will guide treatment decisions, as the majority of the genome still remains undruggable [71]. Essentially, these protocols select for particular subsets of the genome to be subsequently sequenced, a process referred to as target enrichment [72]. For example, for the design of targeted but not personalized assays, recurrent mutations in driver genes from the Catalog of Somatic Mutations in Cancer (COSMIC) may be selected [73].

In terms of assay technology, targeted sequencing can either be amplicon-based or hybrid capture-based. Examples for amplicon-based assays include tagged-amplicon deep sequencing (TAm-Seq) [74], which is now commercially offered by Inivata as Enhanced TAm-Seq [75], Safe-Sequencing System (Safe-SeqS) [76] and Simple, Multiplexed, PCR-based barcoding of DNA for Sensitive mutation detection using Sequencing (SiMSen-seq) [77, 78]. With hybrid capture-based targeted sequencing, select regions within the library are captured using long, biotinylated oligonucleotide baits, or probes. These biotinylated baits have been designed to hybridize to regions of interest (e.g., cancer-related genes, exonic regions) within the fragmented cfDNA and streptavidin is subsequently used to separate the baits bound to target DNA from other fragments which were not bound. An example of this is the pan-cancer AVENIO ctDNA Expanded liquid biopsy panel from Roche—the commercial adaptation of CAncer Personalized Profiling by deep Sequencing (CAPP-Seq) [79]—which contains 17 biomarkers in the U.S. National Comprehensive Cancer Network (NCCN) and other guidelines in addition to 60 biomarkers currently being investigated in clinical trials. Several of these targeted sequencing assays represent what is known as comprehensive genomic profiling (CGP) (Fig. 2A), which refers to a single assay that can detect all major classes of genomic alterations known to drive cancer growth: base substitutions, insertions and deletions, somatic copy number alterations (SCNA) and structural rearrangements.

The largest “panel” that can be applied to cfDNA is WES, i.e., targeting all protein coding genes of the human genome. Indeed, the first landmark studies applying WES to plasma DNA demonstrated that acquired resistance to cancer therapy and disease monitoring is possible in patients with breast cancer [80, 81].

As the development and validation of in-house CGP cfDNA assays require a considerable amount of effort and resources, it simply is neither realistic nor feasible for many labs to design their own comprehensive multi-gene approaches [52] (Fig. 2C). Researchers at the Memorial Sloan Kettering hospital have been pioneers in this field and their recently developed liquid biopsy MSK-ACCESS (Analysis of Circulating Cell- free DNA to Evaluate Somatic Status) assay [32, 82] has been approved by the New York State Department of Health. However, many labs rely on commercial kits to provide in-house end-to-end molecular profiling workflows. Clinics that do not have access to local NGS-based molecular profiling from liquid biopsy through an academic partner may seek solutions from commercial end-to-end providers (Fig. 2C). With this approach, clinicians carry out sample acquisition and shipping in accordance with the vendor’s guidelines and receive a report of molecular findings in return, although reimbursement of such testing approaches is not universal and the service often does not include interpretation or consultation of the findings. Examples for both in-house and service provider solutions include the AVENIO ctDNA panels (Roche; in-house solution), Oncomine™ Pan-Cancer Cell-Free Assay (ThermoFisher; in-house solution), TruSight Oncology 500 ctDNA (Illumina; in-house solution), FoundationOne® Liquid CDx (Foundation Medicine; end-to-end commercial provider) Guardant360™ (Guardant Health; end-to-end commercial provider), Tempus xF Liquid Biopsy Assay (Tempus; end-to-end commercial provider), and elio™ Plasma Resolve (PGDx; end-to-end commercial provider). Many of these CGP solutions offer supplementary and clinically important biomarker information, such as tumor fraction in plasma, estimations of tumor mutational burden (TMB) and microsatellite instability (MSI) status. Industry offerings of CGP solutions for treatment selection in the advanced disease settings are plentiful. However, the SEQC2 Working Group recently reported that when using these kits, the detection of variants had a limited reliability below an allele fraction of 0.5% [83]. Hence, other strategies, such as personalizing assays, are needed to increase sensitivity further.

#### Personalized, targeted assays

Personalized ctDNA assays (Fig. 2A,B) have found application particularly in the early disease setting. Typically, WES or WGS is employed on a patient’s tumor or a baseline plasma sample to identify somatic variants that were not found in the germline sample (Fig. 2B). These detected variants can then be used to design patient-specific multiplex assays to track the mutations from ctDNA in a personalized fashion, increasing the sensitivity of variant detection [84]. Personalized panels are thus designed to maximize the number of informative reads generated. One of the first studies targeted a median of 18 somatic variants and classified a plasma sample as ctDNA-positive when at least two of these variants were detected [69]. Several studies have described tumor-guided sequencing panels with up to 20 different variants [45, 74, 79, 85–87]. However, detection of ctDNA can be vastly enhanced by increasing the number of informative targets in an assay. For example, MRDetect is a tumor-informed detection approach particularly for the minimal residual disease (MRD) setting, which leverages the thousands of somatic mutations typically detectable in solid malignancies to detect tumor fractions with a sensitivity as low as 10^− 5^ [48]. Similar resolution limits were achieved with the Integration of Variant Reads (INVAR) pipeline, which also targets thousands of informative reads [88]. As both approaches involve sequencing of large regions, which is prone for the accumulation of sequencing errors, a sophisticated custom-made error suppression solution is needed. Importantly, both approaches put the need for high sequencing depth into perspective because of the many targets being analyzed. Hence, the sensitivity of such multi-target approaches is rather determined by the breadth and less by the coverage (Fig. 2B). Both approaches are very sensitive for the detection of the presence of ctDNA, but they are less sensitive for the detection of any specific site, such as a driver mutational event, which could be informative about potential therapies. This is a limiting factor of these approaches if the objective of the analysis is to search for druggable targets.

Several commercial providers have adopted such a “tumor-informed” approach. For example, Natera received a Breakthrough Device designation by the FDA in 2019 for its Signatera™ assay that uses a patient’s own tumor mutation signature to personalize an assay for the detection of molecular residual disease, for which utility was originally demonstrated for disease surveillance for patients with metastatic breast cancer (mBC) [89]. Similarly, Inivata’s RaDaR™ assay, which tracks a set of up to 48 tumor-specific variants, was also granted Breakthrough Device Designation as an assay for the detection of residual disease. The sensitivity of RaDaR™ was evaluated in a study of 90 patients with stage IA-IIIB non-small cell lung cancer (NSCLC) who were undergoing radical treatment with curative intent [90]. In early 2021, Exact Sciences acquired a worldwide exclusive license to the Targeted Digital Screening (TARDIS) assay, which was shown to guide treatment strategies in patients with early-stage breast cancer, albeit that the clinically relevant diagnostic threshold will likely have to be refined in further studies [45].

As a substantial number of resources must be invested to devise and validate targeted liquid biopsy platforms, the trend seems to hold that novel methodologies are first developed in the academic setting (Fig. 2C) and then scaled and offered by companies who focus their efforts on improving their implementation and expanding their adoption. As such, centralized ctDNA testing offered by commercial providers may change the paradigm of tumor molecular profiling and some predict that the decentralized model, i.e. testing performed at local labs, will in the future be limited to single-locus or small gene panels, whereas large-scale ctDNA assays will primarily be outsourced to central labs [52].

However, despite all of these caveats, confident detection of MRD is possible in plasma DNA. Tumor genotype-informed MRD detection approaches can attain LODs of ≤ 0.01% (Fig. 2B), which makes them preferable for detection of minute amounts of MRD [69, 84, 85, 91].

#### Analysis of SCNAs

For the detection of genome-wide SCNAs from plasma, a shallow coverage of 0.1x suffices to call these aberrations accurately (Fig. 2A), including the identification of focal events [92–94]. Additionally, tumor fraction in plasma can be estimated specifically from shallow WGS (sWGS) data, sometimes referred to as low-pass WGS [17], which is pertinent to the downstream interpretation of detected alterations as well as any lack of detection.

## Current status of clinical use of ctDNA testing in patients with cancer

The overwhelming technical options of liquid biopsy approaches raise the question as to whether their applications ultimately make a difference for patients’ treatment outcomes. For adoption of a biomarker test in clinical care, three criteria, i.e., analytical validity (measures the accuracy, reliability and reproducibility of a test), clinical validity (assesses the ability of a test to divide a population into separate groups with significantly different clinical outcomes), and clinical utility (evaluates whether outcomes are improved for patients who received the test compared with those who did not), were defined [95]. However, a recent, joint review by the American Society of Clinical Oncology (ASCO) and the College of American Pathologists (CAP) came to the conclusion that there is insufficient evidence of clinical validity and utility for the majority of ctDNA assays in routine clinical care [96]. The ASCO-CAP panel recommended that ctDNA testing should be used only within clinical trials. However, since the publication of this statement, increasing evidence of clinical validity and clinical utility of ctDNA testing has been reported (Table 1).

**Table 1:** Summary of main recently completed and ongoing clinical trials employing ctDNA and NGS

Trial/study (Identifier)	Trial type	Tumor type(s)	No. of patients	NGS/ctDNA detection method	Brief study description	Summary of main findings (if available)
GOZILA (UMIN000016343)	Screening study for companion trials	Metastatic and/or unresectable GI and breast cancers; other solid tumors with specific gene alterations	1,687	Guardant360™	To evaluate the utility of circulating tumor DNA (ctDNA) genotyping. Compared trial enrollment using ctDNA vs. tumor tissue sequencing in the same centers and network	ctDNA-based screening significantly shortened the screening turnaround time and improved the trial enrollment rate without compromising treatment efficacy compared with tissue-based screening [97]
COLOMATE (NCT03765736)	Phase 2 umbrella screening trial	Metastatic colorectal cancer (mCRC) patients with progressive disease	500	Guardant360™	To perform ctDNA-based genomic profiling to enable matching to molecularly targeted therapies	Study ongoing
SLLIP (NCT03248089)	Observational	Treatment naïve, metastatic non-squamous NSCLC	182	Guardant360™	To demonstrate the non-inferiority of cfDNA-based versus tumor tissue-based genotyping	The primary objective was met with cfDNA identifying actionable mutations in 46 patients vs. 48 by tissue. ORR and PFS in patients receiving targeted therapy based on tissue or cfDNA were similar to those previously reported. Confirms that cfDNA-based first-line therapy produced outcomes similar to tissue-based testing [98].
TARGET	Feasibility and screening trial	Solid tumors	100	641 cancer-associated-gene panel in a single ctDNA assay	Two-part study divided into Part A, feasibility of the workflow, ctDNA and tumor sequencing validation, formal reporting and setting up the MTB; and Part B, expansion to match patients to clinical trials and therapies in real time	Four patients experienced an objective response, which represents 36% of the treated patients and 4% of the whole included cohort. Overall, TARGET shows the feasibility of using ctDNA to successfully guide a subset of patients to specific treatment regimens in early-phase clinical trials [19].
plasma-MATCH (NCT03182634; EudraCT2015-003735-36; ISRCTN16945804)	Multicenter, multicohort, phase IIA platform	Advanced breast cancer	1034	Digital droplet PCR for PIK3CA, ESR1, HER2, and AKT1 and Guardant360™	To assess the accuracy of ctDNA testing in advanced breast cancer and the ability of ctDNA testing to select patients for mutation-directed therapy	ctDNA testing offered accurate, rapid genotyping and enabled the selection of mutation-directed therapies, with sufficient clinical validity for adoption into routine clinical practice. Demonstrated clinically relevant activity of targeted therapies against rare HER2 and AKT1 mutations. [99]
NILE (NCT03615443)	Prospective, observational	Advanced nonsquamous non–small cell lung cancer	282	Guardant360™	To demonstrate noninferiority of cell-free circulating tumor DNA (cfDNA)-based tumor genotyping compared to tissue-based genotyping to find targetable genomic alterations	cfDNA detected guideline-recommended biomarkers at a rate similar to tissue testing and outcomes based on ctDNA profiling were comparable to previously published targeted therapy outcomes with tissue profiling, even in community-based centers [100].
IMAGE (NCT01939847)	Non-randomized feasibility study	Progressive, metastatic, triple-negative breast cancer	26	FoundationOne® panel on tumor tissue and blood	To evaluate the feasibility of obtaining a new metastatic tissue biopsy by performing tissue NGS and providing molecular tumor board recommendations within 28 days. In addition, ctDNA from plasma ctDNA was evaluated via NGS, although results were not used to match treatments.	The study highlighted the benefits of parallel ctDNA analysis, as challenges were encountered when trying to obtain NGS results from tumor tissue in the desired timeframe and also due to insufficient sampling. Analysis of ctDNA yielded informative results in 92% of the patients [101].
ICT (EudraCT2014-005341-44	Prospective, two-stage phase II	Advanced and refractory carcinoma	24	50-gene hotspot panel (not cfDNA-specific) and shallow whole-genome sequencing	To evaluate the success of a targeted therapy selected by profiling of ctDNA and tissue in patients with advanced and refractory carcinoma	Informative ctDNA results were obtained in 20/24 patients. A potential tumor-specific drug could be matched in 11 patients and 7 patients received a matched treatment based on ctDNA results. No patient reached the primary endpoint of a PFS ratio > 1.2, indicating that more innovative approaches to study design and matching algorithms are necessary to achieve improved patient outcomes [102]
InVisionFirst-Lung	Multicenter prospective clinical validation study	Untreated advanced NSCLC	264	InVisionFirst®-Lung Circulating Tumor DNA Assay	To prospectively examine the application of plasma-based comprehensive genomic profiling (CGP) in untreated, newly diagnosed, advanced-stage non–small-cell lung cancer (NSCLC) compared with CGP using biopsy tissue	Assay demonstrated high concordance with tissue profiling with suitable sensitivity and specificity for single-gene ctDNA assays. ctDNA-based molecular profiling enabled detection of 26% more actionable alterations compared with standard-of-care tissue testing [103]
SOUND	Open, prospective, interventional, non-randomized IVD study	Patients with locally advanced and/or metastasized carcinoma for whom no further evidence-based treatment is established or who have no satisfactory alternative treatments	200	FoundationOne*®*CDx for tissue analysis, FoundationOne*®*Liquid CDx for ctDNA analysis, AVENIO ctDNA Surveillance panel for biomarker monitoring	One of the largest prospective studies in Austria exploring treatment rates and outcomes of CGP-driven targeted treatment in patients with advanced or metastasized cancer. Additionally, the treatment decision process will be supported and documented by the NAVIFY Tumor Board software	Study ongoing
Circulating Tumor DNA (ctDNA) for Early Treatment Response Assessment of Solid Tumors (NCT04354064)	Observational cohort study	Diverse solid tumors	3362	Not given	To enable earlier detection of disease recurrence through analysis of ctDNA from plasma and urine	Study ongoing
COBRA (NCT04068103)	Interventional, randomized phase II/III study	Stage IIA colon cancer	1408	GuardantHealth LUNAR panel	To compare the rate of ctDNA clearance in “ctDNA detected” patients treated with or without adjuvant chemotherapy following resection of stage IIA colon cancer. (Phase II). To compare recurrence-free survival (RFS) in “ctDNA detected” patients treated with or without adjuvant chemotherapy following resection of stage IIA colon cancer. (Phase III)	Study ongoing
*Studies with associated ctDNA analyses although ctDNA not trial focus*						
**Trial/study (Identifier)**	**Trial type**	**Tumor type(s)**	**No. of patient used for ctDNA analysis**	**NGS methods for ctDNA analysis**	**Brief description of ctDNA use**	**Summary of main findings with implications for ctDNA use**
I-PREDICT (NCT02534675)	Cross-institutional, prospective, observational, navigation	Patients with incurable malignancies with aggressive biology	12	FoundationACT® (62-gene panel)	NGS was also performed on ctDNA to extend the possibilities identifying actionable targets and personalizing treatment with combination therapies	Achieved a treatment matching rate of 49% (73 of 149 patients), higher than in other precision medicine trials. This was likely the result of several key factors: Using a large panel of cancer-related genes, including MSI status, PD-L1 IHC and ctDNA results [33]
PALOMA-3 (NCT01942135)	Randomized, double blind, placebo controlled, Phase 3	Hormone receptor + HER2-negative metastatic breast cancer after endocrine failure	459 patients with a baseline plasma sample available, 287 of these having a matched EOT	Whole-exome sequencing and targeted sequencing with a custom 14-gene panel	Plasma ctDNA exome sequencing of paired baseline and EOT samples from 195 patients enrolled on the PALOMA-3 trial was performed to investigate the mechanisms of resistance to the CDK4/CDK6 inhibitor palbociclib plus fulvestrant versus fulvestrant alone	ctDNA results showed that acquired resistance to fulvestrant and palbociclib is associated with clonal evolution and acquired mutations in RB1, PIK3CA, and ESR1. These results highlight the potential of ctDNA to guide the next line of treatment [104]
HERACLES (NTC03225937)	Proof-of-concept, multicenter, open-label, phase II trial	Patients with KRAS exon 2 (codons 12 and 13) wild-type and HER2-positive metastatic colorectal cancer refractory to standard of care	30	Guardant360™ assay	Plasma from patients treated with trastuzumab and lapatinib in the HERACLES study was collected before treatments very 15 days during therapy, and at the time of radiographic progression	Mutations in RAS and BRAF were detected in pretreatment plasma samples and were associated with primary resistance to HER2 treatment. Patients enrolled had received anti-EGFR therapy prior to enrollment, which led to the emergence of RAS-mutant clones. This study suggests that ctDNA may be used to determine patient eligibility for HER2-targeted therapy to spare patients unnecessary treatment [105]
IMvigor010 (NCT02450331)	Phase III, open-label, randomized, multicenter	Patients with high-risk muscle-invasive urothelial carcinoma	581	Whole exome sequencing of tumor tissue followed by personalized Signatera™ ctDNA assay	Evaluated outcomes in patients who had undergone surgery and were evaluable for ctDNA from a randomized phase III trial of adjuvant atezolizumab versus observation in operable urothelial cancer	ctDNA testing at the start of therapy (cycle 1 day 1) identified 214 (37%) patients who were positive for ctDNA and who had poor prognosis. ctDNA-positive patients had improved DFS and OS in the atezolizumab arm versus the observation arm. Data suggest that adjuvant atezolizumab may be associated with improved outcomes compared with observation in patients who are ctDNA-positive and at a high risk of relapse, which could shift approaches to postoperative care [106]

In the clinic, ctDNA analysis is emerging as a biomarker for three different scenarios: first, as a prognostic biomarker across multiple cancer types; second, for response assessment; and third, for resistance monitoring, i.e., identification of disease progression during or after systemic therapy ahead of clinical or radiographic indicators.

This development is reflected in key recommendations from the NCCN guidelines, which were recently reviewed [107]. In brief, NSCLC has evolved to the tumor type with the most compelling and comprehensive evidence for ctDNA testing. The NCCN Guidelines for NSCLC (version 7.2021) (https://www.nccn.org/guidelines/guidelines-detail?category=1&id=1450) recommend molecular testing at the time of diagnosis, with repeat biopsy or plasma testing to enable identification of genomic alterations in *EGFR*, *ALK*, *ROS1*, *BRAF*, *MET*, and *RET* to guide the use of US Food and Drug Administration (FDA)-approved targeted therapies in the first-line advanced disease setting. Two recent studies confirmed that plasma ctDNA NGS in advanced NSCLC can increase the positive identification of guideline-recommended genomic biomarkers and actionable alterations [100, 103]. The cobas® EGFR mutation test v2 was the first liquid biopsy assay approved by the FDA [108] as a companion diagnostic test for screening *EGFR* mutations from plasma cfDNA.

Furthermore, a current challenge is the identification of patients with NSCLC who may achieve durable benefit from immune checkpoint inhibitor (ICI) treatment. On-treatment changes of ctDNA in plasma have been shown to reveal predictive information for long-term clinical benefit in ICI-treated patients and molecular ctDNA responses correlated with radiographic response to ICI [35, 109–112]. A multi-parameter model that integrates ctDNA levels with circulating immune cells, which mirror the immune milieu, may further improve prediction of tumor response to ICI treatment [113].

Another key cancer type with compelling evidence that ctDNA testing provides clinically relevant information is breast cancer. Approximately 40% of hormone receptor (HR)-positive, human epidermal growth factor receptor 2 (HER2)-negative breast cancers carry *PIK3CA* mutations. Identification of these *PIK3CA* mutations are informative about treatment with the PI3Kα-specific inhibitor alpelisib in combination with fulvestrant as second-line therapy for advanced disease [114, 115] and, accordingly, the NCCN guidelines for invasive breast cancer (version 1.2022) (https://www.nccn.org/guidelines/guidelines-detail?category=1&id=1419) recommend assessment for *PIK3CA* mutations using tumor tissue or ctDNA testing, with reflex tumor testing if ctDNA results are negative. Indeed, plasma-based reassessing of *PIK3CA* status is important, as *PIK3CA* mutational status can change upon disease recurrence [25, 78]. On the basis of data from the phase III SOLAR-1 trial [114], the therascreen® PIK3CA RGQ PCR kit was granted FDA approval for the detection of *PIK3CA* mutations in plasma or tumor tissue in patients with advanced-stage (HR)+/HER2 − breast cancer. Further examples are patients with metastatic, ER-positive breast cancer who progressed on endocrine therapies. Variants in *ESR1* become much more prevalent in mBC, indicating that the presence of these mutations arise because of the evolving cancer. Importantly, ctDNA analysis can non-invasively detect *ESR1* mutations that herald resistance to aromatase inhibitors to tailor adjuvant therapies [116].

For most other tumor entities, the NCCN guidelines do not directly address plasma ctDNA testing but acknowledge that relevant genomic alterations may be identified by evaluating ctDNA in the blood for a variety of cancers [107]. However, there are compelling near-term emerging applications for ctDNA analysis, in particular for patients with prostate cancer. The PARP inhibitor (PARPi) olaparib was FDA-approved for patients with metastatic, castration-resistant prostate cancer (CRPC) with deleterious or suspected deleterious germline or somatic homologous recombination repair (HRR) gene mutations [117]. For patients with metastatic CRPC (mCRPC) and deleterious germline and/or somatic *BRCA* mutations, rucaparib received approval [118]. Plasma ctDNA testing will be useful to identify the subset of patients eligible for these treatment approaches. Furthermore, ctDNA testing has been applied to understand primary resistance to abiraterone and enzalutamide [119–121] as well as studying molecular alterations involved in neuroendocrine transformation [122].

Importantly, several seminal studies have demonstrated the prognostic value of ctDNA MRD detection and in doing so have proven the clinical validity of these strategies. A recent meta-analysis of these studies came to the conclusion that, following definitive therapy for solid cancers, ctDNA MRD testing is strongly prognostic and has high positive-predictive value for risk of occurrence [84]. The clinical sensitivity, i.e., the percentage of patients with recurrent disease and who were ctDNA-positive after therapy, approached 100% in most studies when a surveillance strategy, i.e., evaluation of multiple posttreatment blood draws during follow-up, was conducted [84]. Another important aspect of ctDNA MRD testing is that residual disease was identified with a lead time of several months earlier than by standard-of-care radiological imaging [84].

However, while the clinical validity of ctDNA MRD testing is clearly established, there is less evidence for its clinical utility, i.e., demonstration of a benefit from early initiation of additional therapy after ctDNA MRD detection. One study conducted with patients with locally advanced NSCLC who were ctDNA MRD-positive after chemoradiation and who received consolidation immunotherapy demonstrated that these patients had significantly better freedom from progression than patients treated with chemoradiation alone [111]. Another study showed that adjuvant atezolizumab in muscle-invasive urothelial cancer might improve survival in ctDNA-positive patients [106]. These two studies provided the first evidence that personalized therapy based on ctDNA MRD status may result in improved patient outcomes. In summary, regarding ctDNA MRD testing, there is clear evidence for clinical validity and emerging signs for clinical utility.

### Real-world ctDNA use cases for advanced cancer

**Fig. 3:**
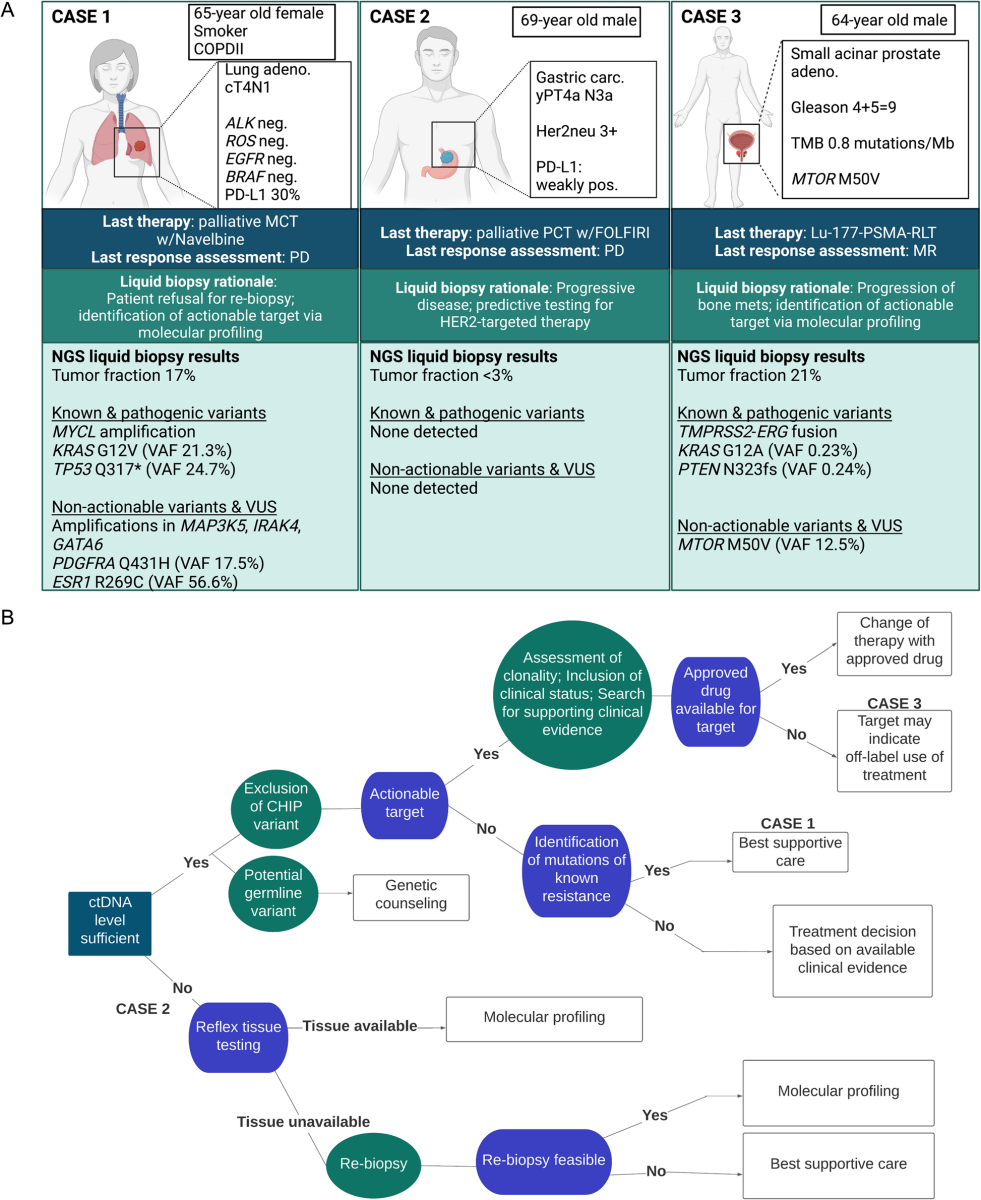
Use cases for ctDNA analysis throughout the cancer patient journey: identification of actionable targets in patients with advanced cancer. **A** Representation of 3 real-world cases of patients with confirmed progressive disease where liquid biopsy was justified to identify actionable targets. Available clinical patient characteristics and primary tumor biopsy profiling data are displayed in the white box. The last received therapy along with the associated measured radiological response (RECIST 1.1) are in the dark blue box and the specific rationale for ordering a liquid biopsy is listed in the dark green box. Below, a summary of the NGS results from comprehensive genomic profiling (CGP) via the AVENIO ctDNA Expanded Panel are documented, including: tumor fraction in plasma estimated via ichorCNA (%; LOD 3%); clinically relevant and pathogenic somatic copy number alterations (SCNAs) and variants, with variant allele frequency (VAF, %) detected from plasma DNA; non-actionable and variants of unknown significance (VUS). Of particular note is Case 3, which had a relatively high tumor fraction of 21%, but the two pathogenic variants detected had VAFs <1%. As these low allele fractions (KRAS G12A: 0.23%, PTEN N323fs: 0.24%) do not align with the overall tumor content of the sample, these VAFs may indicate subclonality of the alterations or potential sequencing artifacts. For this reason, it would be necessary to confirm their presence with an orthogonal approach using a new blood sample, especially if they were to influence a treatment decision. **B** Basic decision tree for this use case and the interpretation of detected alterations from liquid biopsy NGS data. The cases in (**A**) are mapped at the corresponding position that reflects the individual scenario. The critical starting point is the assessment of ctDNA level, i.e. tumor fraction (TF), in plasma, as samples with sufficiently low TF may not yield any detected alterations (Case 2). In such cases, reflex tissue testing is the clinical standard. If the sample has sufficient a ctDNA level, the analyst must rule out potential CHIP or germline variants before moving on to actionability assessment. In some cases, mutations associated with resistance are detected (Case 1), but no therapeutic targets are found. The identification of actionable targets and matching of potential suitable, evidence-based treatments is not a straightforward process and thus should be discussed at a molecular tumor board with oncologists to derive the final treatment decision (Case 3)

**Fig. 4:**
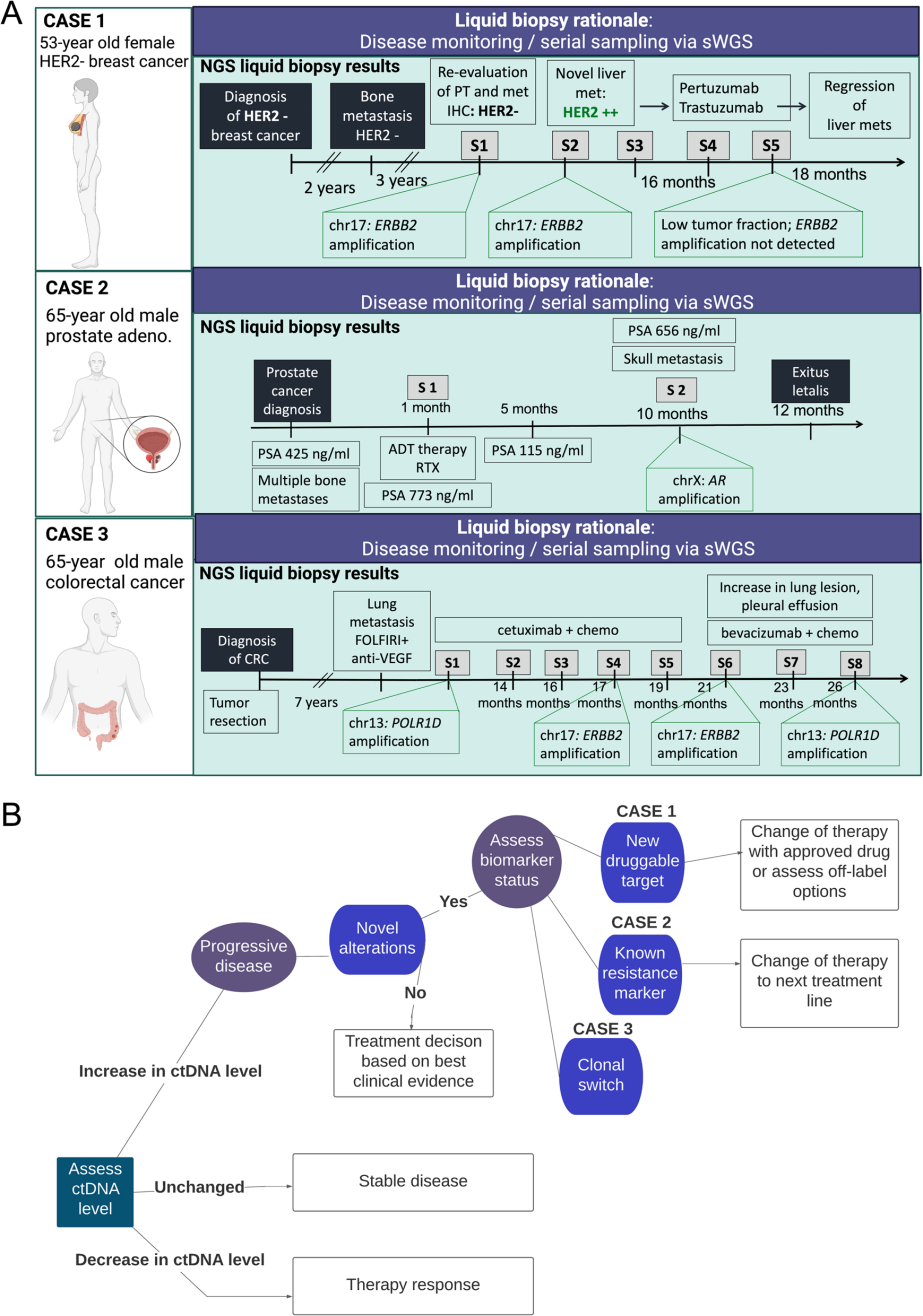
Use cases for ctDNA analysis throughout the cancer patient journey: disease monitoring. **A** Representation of 3 real-world cases of patients who underwent serial liquid biopsy sampling for disease monitoring purposes via shallow whole-genome sequencing (sWGS). Patient age and tumor entity are displayed in the white box. In the green panels, the NGS results from sWGS monitoring are shown in patient timelines. The serial samples are listed in the gray boxes (e.g. S1, S2, etc.). Detected focal somatic copy number alterations (SCNAs) are shown in the green callout boxes at the corresponding time point that they were detected via sWGS.**B** Basic decision tree for this use case and the interpretation of detected alterations from disease monitoring data via liquid biopsy. The cases in (**A**) are mapped at the corresponding position that reflects the individual scenario. Again, assessment of the ctDNA level in plasma represents the critical first step, as decreases in ctDNA from the previous sample may indicate a response to therapy, whereas unchanged levels may indicate stable disease. Increases in ctDNA fraction are generally associated with progressive disease. In some cases, novel alterations may be detected via monitoring and may represent novel druggable targets that were not observed from previous profiling (Case 1), known resistance markers (Case 2), or a clonal switch, which demonstrates the adaptive nature of tumors under the selective pressure of targeted therapies (Case 3)

In parallel to the numerous ongoing efforts striving to prove the clinical validity and utility of ctDNA-based testing as described (Table 1), the fact is that molecular profiling of plasma DNA is already a reality for many in academic clinical settings who treat patients with advanced disease. Although tissue biopsy remains the gold standard of cancer diagnosis and represents the primary analyte for guiding treatment decisions, it is not at all uncommon that tumor tissue is simply not available. For example, a patient with clinically confirmed progressive disease who has exhausted all standard lines of treatment may be asked to undergo re-biopsy for retrieval of the latest tumor signal from tissue to derive the next decision for therapy. However, patients may either refuse re-biopsy or may not qualify as candidates for such a procedure. Rather than skipping molecular profiling altogether, here, the analysis of ctDNA may serve as a tumor-agnostic surrogate analyte to detect potential actionable targets. In several cohorts, including prospective studies, large NGS panels have proven advantageous in the detection of actionable targets from ctDNA [19, 30, 98, 99, 102, 123], which is also reflected in the wide assortment of industry offerings of CGP solutions for treatment selection in the advanced disease setting. We have summarized the indication, liquid biopsy rationale, and NGS data from three real-world cases of patients who underwent profiling to identify actionable targets (Fig. 3A). As with any biological data that is unique to the individual, interpretation of the alterations detected and subsequent clinical decisions are not always clear-cut, but may be simplified for exemplary purposes in the form of a decision tree (Fig. 3B). Here, it is important to emphasize that variant interpretation is an intricate process, requiring molecular expertise and at present not entirely standardized [124–126]. As such, clinical decisions derived from combined genomic and immunohistochemistry data must be discussed within the framework of an experienced MTB. Similarly, in the advanced disease setting, patients enrolled in liquid biopsy programs may undergo serial sampling to monitor their treatment. In certain scenarios, real-time treatment monitoring via ctDNA analysis may uncover a novel actionable target before it is detected via tissue biopsy or may provide evidence for the development of a novel resistance-related event (Fig. 4A), both which indicate the potential for a change in the course of treatment (Fig. 4B).

### Confounding factors and limitations of ctDNA assays

In order to properly obtain and preserve rare DNA fragments from the circulation, efforts have been dedicated to determining proper pre-analytical workflows crucial to reliable downstream cfDNA analysis, such as blood collection and sample transport, centrifugation, storage and isolation methods [60, 70, 127], as well as technical aspects of the detection of aberrations [55, 128].

To date, a fundamental understanding regarding the biological mechanisms behind release and clearance of cfDNA is still lacking, although this topic is increasingly being explored [129–132]. Levels of normal cfDNA may also increase due to non-malignant conditions, such as tissue injury or inflammation. In particular, after tumor removal, post-surgical inflammatory changes may cause an increase in cfDNA levels postoperatively for several weeks and hence dilute the allele fraction of ctDNA molecules [133]. Hence, increased plasma DNA levels cannot generally be equated with increased ctDNA levels in patients with cancer.

The low ctDNA fractions pose several challenges and detailed knowledge about the limit of detection (LOD) of the selected assay is vital [84]. The LOD depends on both biological and technical factors. Early landmark studies have described the variable levels of ctDNA in diverse solid tumors and across stages that correlate with tumor burden [24] and demonstrated a half-life of ctDNA ranging from several minutes to several hours [134], although proper pharmacokinetic studies to accurately determine this have not yet been performed. Biological factors determine the tumor DNA shedding rate. Indeed, not every tumor type “sheds” its DNA equally into the bloodstream, which influences the measurable quantity of ctDNA [24, 70]. Biological factors associated with tumor DNA shedding include tumor volume and tumor surface area, vascularization, tumor cell growth and death rates, mitotic and metabolic activity, and cell morphology. Furthermore, cancer signal detection is associated with active proliferation and explains why more aggressive cancers tend to shed more DNA into the bloodstream [135]. In fact, longitudinal follow-up data to evaluate the prognostic significance of a multi-cancer early detection (MCED) test has suggested that this test detected more clinically significant cancers and that detection was prognostic beyond clinical stage and method of clinical diagnosis. Accordingly, cancers not detected by the MCED test tended to be less aggressive [135]. Hence, biological differences in shedding rates may explain the differences in sensitivity between various tumors and high ctDNA levels may be an indication for more aggressive cancers.

Somatic variants from non-tumor tissues represent another biological confounding factor and mainly relate to the prevalence of CHIP (clonal hematopoiesis of indeterminate potential) derived mutations in ctDNA, i.e., normal hematopoietic cells accumulating somatic mutations during the aging process in the absence of cancer [136–138]. CHIP is highly prevalent in the general population and these mutations from hematopoietic cells may be misinterpreted as tumor-derived in cfDNA analysis. In fact, a majority of cfDNA mutations may be derived from clonal hematopoiesis, making matched cfDNA-white blood cell sequencing mandatory for accurate variant interpretation [27, 35, 82], albeit not yet a universally adopted practice due to the associated increased costs of sequencing an additional sample per patient.

Importantly, commercial products should not be considered a panacea, as their actual performance capabilities have yet to be critically tested in large, multicenter studies. As mentioned above, their reliability below an allele fraction of 0.5% is likely limited [83]. Furthermore, numerous published studies involve black box chemistry and complex bioinformatics, which are hard to comprehend or to verify, even for experts in the field. As a consequence, assessment of reproducibility and sensitivity by second non-profit-based parties is often lacking. At the same time, the innovative bioinformatics algorithms developed for WGS analysis are by no means self-explanatory and require a deeper intersection of basic biological and technical knowledge. Some studies, which are based on use of machine learning classifiers, may be prone to over-performance, even if they used separate training and validation cohorts, such that many of the published models require validation in prospective, multi-center studies.

A detailed discussion of technical errors, which occur *ex vivo* during the various molecular biology steps, leading to artificial mutations, is beyond the scope of this review and has been reviewed previously [50, 84].

### Breakdown of applications in the pipeline

**Fig. 5:**
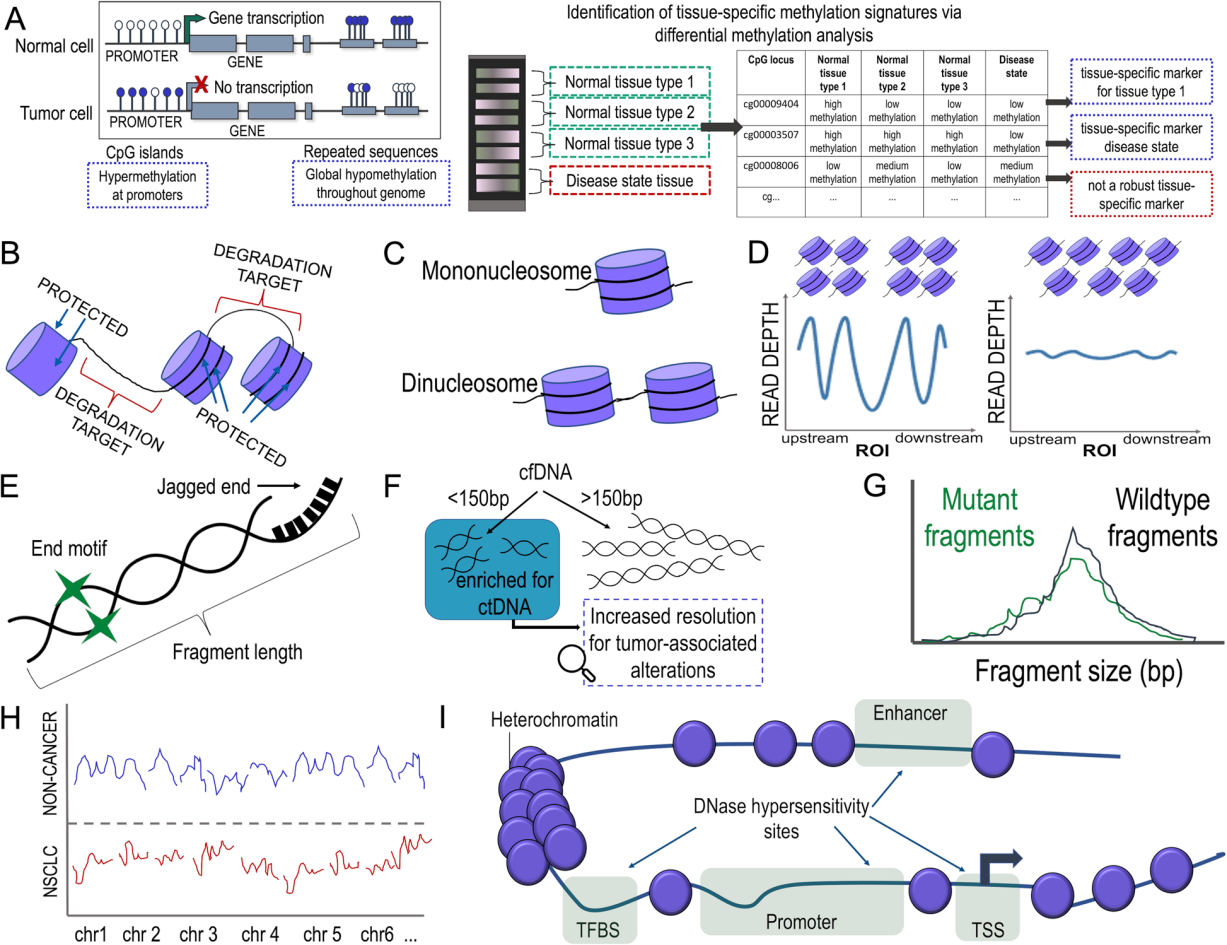
Methylation analysis and whole-genome sequencing of cfDNA enables applications that extend beyond DNA sequence and copy number. **A** (Left) Methylation patterns of DNA in tumor cells may look different from their normal, healthy counterparts. Generally, CpG islands are associated with promoter regions of genes and these regions are prone to hypermethylation, i.e., gain of methylation, in tumor cells, leading to a block of gene transcription, as the bulky transcription machinery is prohibited from binding to the hypermethylated site. Conversely, tumor cells exhibit a general trend of global hypomethylation, i.e., loss of methylation, throughout the genome, which is frequently observed at repetitive sequences. The lollipops represent CpG sites, with white lollipops indicating no methylation at this particular cytosine and dark blue representing methylation at the cytosine. (Right) Typically, beadchip array data can be harvested to perform differential methylation analysis between various tissue types of interest, e.g. comparing normal breast tissue and malignant breast tissue or identifying differences in methylation between healthy colon or lung tissue. Differential individual CpGs or regions of differential methylation can be identified for use as a tissue-specific marker for downstream purposes. CpGs or regions of CpGs that do not confer a highly differential methylation signal from other analyzed tissues will not constitute a robust tissue-specific marker. **B** Apoptotic death of cells results in the digestion of open chromatin, i.e. regions of DNA not bound to and protected by nucleosomes. Naked DNA not associated with proteins, e.g., histones or TFs, will be digested and not detected in the circulation. **C** The majority of cfDNA is thus mononucloeosomal DNA. However, longer fragments of DNA may be protected by two nucleosomes, i.e. dinucleosome. **D** The coverage patterns of where the reads align in the genome reflect the biology of that particular region. The coverage patterns at regions of interest (ROI) reflect the original positioning of nucleosomes in cells. Generally, well-defined nucleosome organization and positioning in cancer cells may indicate that the ROI is “open” or accessible. This is accompanied by a drop in coverage at the ROI, where no nucleosomes were positioned, resulting in what is referred to as the nucleosome-depleted region (NDR). Densely packed nucleosomes with less defined positioning reflects that the region is not accessible or “closed”, with no drop in coverage at the NDR and no oscillation of coverage upstream or downstream to the ROI. Example ROIs are transcription start sites (TSS), transcription factor binding sites (TFBSs), or DNase hypersensitivity sites (DHS). **E** The types of fragment features that can be observed are diverse, such that there is no one-size-fits-all approach to applying fragmentomics to cfDNA. Exemplary features that can be harvested for analysis are illustrated on this DNA strand, including fragment length. Green stars represent that plasma DNA ends show prevalence of certain nucleotide contexts, i.e., preferred fragment end motifs, which are defined as a few nucleotides at plasma DNA ends regardless of the site of origin within the genome. Detection of double-stranded plasma molecules carrying single-stranded protruding ends are termed jagged ends, which may be harnessed to assess the jaggedness across varying plasma DNA fragment sizes and their association with nucleosomal patterns. **F** Because ctDNA has a modal size profile shorter than that of the background cfDNA originating from non-cancerous cells, this fragment size feature can be used to enhance detection of tumor-associated alterations. Shorter fragments of cfDNA can be harvested either through specialized library preparation approaches that enrich for short cfDNA molecules, through *in silico *size selection approaches, or a combination of both. **G** Fragment size differences have also been shown to differentiate between mutations stemming from CHIP and those originating from the tumor. CHIP-associated mutations are associated with fragment size distributions of wildtype molecules (black distribution), whereas tumor-associated mutations typically reside on short cfDNA fragments (green distribution). **H** Using WGS data, global fragmentation patterns can be observed. By establishing coverage and size distribution references of cfDNA fragments in defined genomic windows in both healthy and cancer populations, it can be determined whether an individual’s cfDNA distribution is likely to be healthy (blue signal) or cancer-derived (red signal). By comparing genome-wide profiles between various tissues, these patterns may also be used for tissue deconvolution purposes. **I** Nucleosomes (purple circles) are shown in the form of heterochromatin or open chromatin regions along a length of DNA. Open chromatin consists of regulatory regions within the genome, such as enhancers, transcription factor binding sites (TFBS), promoters, and transcription start sites (TSSs), to which proteins may bind. These are highlighted in green and collectively represent DNase hypersensitivity sites (DHS). When a canonical nucleosome is supplanted, the underlying DNA is rendered accessible to nucleases and other protein factors

So far, the ctDNA assays described herein have focused on somatic tumor-associated mutations and copy number alterations. However, epigenetic alterations, such as methylation markers, cfDNA fragment length, and open chromatin regions, will increasingly take on an important role [139, 140]. Tissue-specific, stable, and universal methylation patterns can be used to detect cell death and to monitor even common diseases, such as inflammation, cardiomyocyte cell death or pancreatitis with cfDNA testing (Fig. 5A). These epigenetic differences can be leveraged in cfDNA analysis to determine the exact origin of cfDNA, which is referred to as plasma DNA tissue mapping or plasma DNA tissue deconvolution (Fig. 5A). To date, most plasma DNA tissue mapping studies have been conducted using methylation markers [11, 141–145]. Furthermore, the MCED tests mentioned above have been based on such methylation markers and have described the option of detecting more than 50 different cancer types [135, 146]. Early detection of cancer is at present a very active area of research and other efforts have used a combination of mutations and circulating proteins [147, 148]. These tests offer the option of detecting a range of cancers early, which may reduce cancer-related death. At present, their feasibility as a screening test in healthy populations is being tested with large-scale prospective studies, such as Grail’s The Circulating Cell-free Genome Atlas Study (CCGA; NCT02889978) or STRIVE trial (NCT03085888) as well as Thrive’s ASCEND trial (NCT04213326), all of which already began before 2020. Several of such early detection efforts have recently been reviewed [149].

In general, typical cfDNA fragment lengths after enzymatic processing in apoptotic cells have a modal distribution of 166 bp, a size that corresponds approximately to the length of DNA wrapped around a nucleosome (∼147 bp) and a linker fragment (∼20 bp) [150–152]. The nucleosome protects the DNA from enzymatic digestion in apoptotic cells so that DNA is mainly degraded in the intervening linker fragments (Fig. 5B). As a result, cfDNA consists mostly of mononucleosomal DNA, but dinucleosomal cfDNA fragments may also be observed (Fig. 5C). Several studies have provided evidence that cfDNA indeed reflects such nucleosome footprints [122, 153, 154]. Coverage-based analysis can be used for nucleosome position mapping (Fig. 5D), but other approaches for nucleosome position mapping have also been described [122, 145, 153]. The strong impact of the cellular nucleosomal organization on the DNA fragmentation patterns [29, 155–158] results in characteristic signatures regarding fragment size and nucleotide motifs including—in addition to the fragment length—end-motif frequency or jagged ends (Fig. 5E). Applications of special protocols may reveal the presence of cfDNA fragments that deviate from the canonical cfDNA sizes. Single-stranded DNA sequencing revealed the existence of ultrashort cfDNA (~ 50 bp) [159], whereas single-molecule sequencing found a population of long (up to ~ 23,000 bp) cfDNA molecules [160]. Such molecules at the extreme spectrum cfDNA fragment lengths may open up new clinical applications. Altogether, this novel and very evolving area in liquid biopsy research is referred to as “fragmentomics” [161, 162] (Table 2).

**Table 2:** Exemplary approaches harnessing fragmentomics, potential applications and limitations

Fragmentomics feature of interest	Description	Main/potential applications	Approach limitations	Reference
Windowed protection score (WPS)	Whole-genome sequencing to generate maps of genome-wide nucleosome occupancy; WPS is calculated by the number of DNA fragments completely spanning a 120 bp window centered at given genomic coordinate, minus number of fragments with an endpoint within that same window	Use of nucleosome footprints can infer cell types contributing to cfDNA; use of short cfDNA fragments to footprint TFs	Nucleosome maps are heterogeneous, comprising signals of all cell types that give rise to cfDNA; profiled only a small number of ubiquitous TFs; small size of reference dataset of cell lines and tissues against which these samples were compared	[153]
Fragment coverage	Whole-genome sequencing of plasma DNA identified two discrete regions at TSS (NDR and 2K region) where nucleosome occupancy results in different read depth coverage patterns for expressed and silent genes	Classification of expressed cancer driver genes; Determination of expressed isoform of genes with several TSSs	High tumor fraction in plasma required; Gene expression prediction limited to amplified regions; binary classification of genes, i.e. expressed vs. non-expressed	[94]
Fragment coverage	Establishment of nucleosome occupancy maps at TFBSs via whole-genome sequencing; Calculation of accessibility scores as a measure of strength of nucleosome phasing at binding sites of a TF, reflecting strength of TF binding	Identification of lineage-specific TFs and profiling of individual TFs from cfDNA; Identification of patient-specific and tumor-specific patterns, including prediction of tumor subtypes in prostate cancer; detection of early-stage colorectal carcinomas	TF nucleosome interaction maps are heterogeneous, comprising signals of all cell types that give rise to cfDNA; use of all 504 TFs in logistic regression model does not make strategy specific for colon cancer; further work required to identify distinct TFs subsets specific for different tumor types	[122]
Incorporation of additional information on DNA fragment lengths and tumor allelic fraction of mutations to enhance the accuracy of ctDNA detection	Analysis of DNA fragment sizes in plasma cfDNA from melanoma patients demonstrated that mutant fragments were shorter than wild-type fragments at the mononucleosome and dinucleosome peaks; Assessed frequency of mutations for any given fragment size and then weighted each mutant read observed with the probability that it came from the cancer distribution as opposed to the wild-type size distribution	Personalized cancer monitoring	Not suitable for early detection or diagnosis of new cancers, as it requires evaluation of signals across a patient-specific list of mutations; only applied to limited number of cases; lack of validation in a larger cohort	[88]
Filtration of CHIP-associated variants according to fragment size	Demonstrated that cfDNA molecules bearing CH-derived variants tend to be longer than those bearing tumor-derived variants, which can be leveraged to improve detection sensitivity of ctDNA	Early-stage lung cancer detection	Larger cohort needed to fully establish performance characteristics of Lung-CLiP; majority of cases were incidentally diagnosed lung cancers and not identified by LDCT screening; cohort mainly composed of smokers and thus need to assess performance in non-smokers	[27]
Global fragmentation patterns	Evaluation of size distribution and frequency of millions of naturally occurring cfDNA fragments across genome	Non-invasive early detection/prescreening high-risk populations for lung cancer; Use of genome-wide fragmentation profiles across ASCL1 TFBSs to distinguish individuals with SCLC from those with NSCLC	Majority of patients in LUCAS cohort presented symptoms not fully representative of a screening population; lack of large prospective validation in a screening population; Several patients with late-stage disease not detected by DELFI	[29]
Orientation-aware cfDNA fragmentation (OCF)	Assessment of differences in read densities of sequences corresponding to orientation of upstream and downstream ends of cfDNA molecules in relation to reference genome; quantitative analyses of signals to measure relative contributions of various tissues to plasma DNA pool	Noninvasive prenatal testing, organ transplantation monitoring, cancer liquid biopsy	Only small sample size investigated; method based on open chromatin profiles, of which availability was limited at the time of the study	[145]
Preferred end coordinates	Determined that particular genome coordinates had an increased probability of being an ending position for plasma DNA fragment and whether such ends exhibit differences depending on their tissue of origin (i.e., from placenta or mother or from patients with HCC)	Noninvasive fetal whole-genome analysis; diagnosis of early-stage HCC; tissue-of-origin for organ transplant recipients	Determination of preferred-end sites requires high coverage sequencing; lack of large prospective validation cohort	[163, 164]
DNA end motif	Demonstrated that plasma DNA ends show prevalence of certain nucleotide contexts, i.e., preferred fragment end motifs, which represent a distinct type of fragmentation signature. The motifs are defined as a few nucleotides at plasma DNA ends regardless of the site of origin within the genome	End motifs may serve as class of biomarkers for liquid biopsy in oncology, noninvasive prenatal testing, and transplantation monitoring	Small sample size; lack of large-scale validation study	[165]
Jagged ends	Detection of double-stranded plasma molecules carrying single-stranded protruding ends, termed jagged end; Assessment of jaggedness across varying plasma DNA fragment sizes and association with nucleosomal patterns	Fragmentomics-based molecular diagnostics in noninvasive prenatal testing, organ transplantation, oncology, and autoimmune diseases	Lack of large-scale validation study	[166]
Global and regional fragment size distribution, fragment coverage (LIQUORICE)	Combination of several fragmentation-based metrics into an integrated machine learning classifier; Analysis of global fragment size distribution, region fragment size distribution as well as fragment coverage at regions of interest	Use of cfDNA fragmentation patterns as prognostic biomarkers in Ewing sarcoma	Lack of standard reference markers for ctDNA quantification makes calculation of definitive performance metrics for machine learning classifier difficult; investigation of rare sarcomas, which limited size of cohort; retrospective analysis lack of validation in large, prospective cohort	[167]

Importantly, several studies have demonstrated that ctDNA has a modal size profile shorter than that of the background cfDNA originating from non-cancerous cells [156, 168]. To date, there are many efforts to explore how this knowledge can be leveraged to employ fragment size analysis to enhance detection of ctDNA [156]. For example, it has been shown that focusing on shorter cfDNA fragments enhances the detection of ctDNA [156] (Fig. 5F). Another important application is to use these size differences to distinguish between CHIP-associated mutations, which usually reside on cfDNA fragments with a size distribution of non-cancerous molecules, whereas mutations present in matched tumor specimen are more frequently on significantly shorter cfDNA molecules (Fig. 5G) [27]. Furthermore, machine learning models have been developed for detecting tumor-derived cfDNA through genome-wide analyses of cfDNA fragmentation in individuals at risk for lung cancer, suggesting that global fragmentation profiles may emerge as a tool for non-invasive detection of lung cancer [29, 157] (Fig. 5H). Importantly, as in any cfDNA application, pre-analytical processing methods must be carefully considered when performing fragmentomics-based analyses [159].

Open chromatin regions refer to regulatory regions within the genome, such as promoters, enhancers, or silencers (Fig. 5I), to which proteins bind, supplanting a canonical nucleosome and rendering the underlying DNA accessible to nucleases and other protein factors (Fig. 5I). As these are often regulatory regions with a biological role in tissue differentiation [169], nucleosome patterns and open chromatin regions are—similar to methylation markers—highly tissue-specific and can be also be used for plasma DNA tissue mapping and even tumor subtyping [122, 153]. Furthermore, plasma DNA-deduced nucleosome maps have been shown to result in characteristic coverage densities at transcription start sites, which correlate with gene expression from cells releasing their DNA into the circulation [154]. Similar patterns can also be observed at transcription factor binding sites (TFBS) and correlate with the accessibility of these transcription factors such that deregulation of transcription factors, an important driver in tumorigenesis, can be inferred from cfDNA [122]. Another application demonstrated that tissue-specific cfDNA degradation and nucleosome patterns enable the quantification of ctDNA burden [170] as well as for the detection and classification of pediatric solid tumors [167].

In summary, the detailed analyses of these epigenetic cfDNA features represents a novel, emerging area that will enable vital biological insights into the process of DNA release into the circulation [162] as well as more detailed clinically relevant characteristics about the tumor genome that are not attainable by the mere investigation of mutations or SCNAs.

**Fig. 6:**
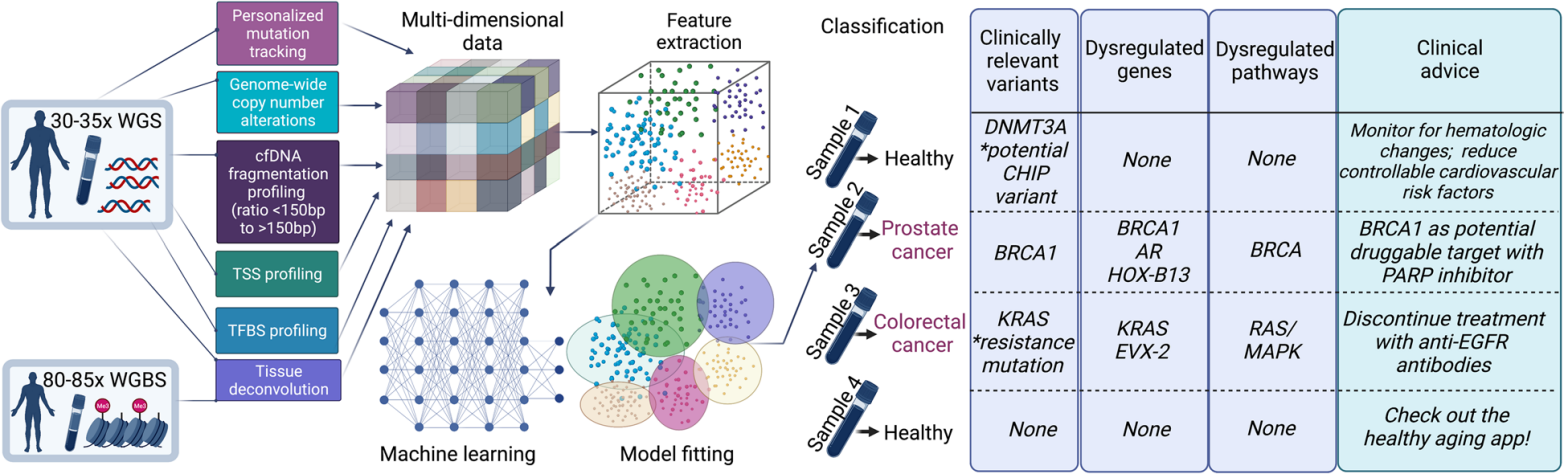
A personal viewpoint on future plasma DNA analyses: one single dataset, an impressive number of analysis opportunities from cfDNA. In the future, there is little doubt that we will be sequencing whole patient genomes. Although current precision cancer medicine programs predominantly rely on gene panel sequencing, the decreasing cost of WGS will soon provide an attractive alternative, replacing stand-alone cancer diagnostics tests that require separate validation and standardization procedures. From a moderate sequencing coverage of 30-35x, we will be able to harvest cfDNA information from a single dataset, encompassing analysis possibilities ranging from personalized mutation tracking to tissue deconvolution. However, as methylation markers serve as the current predominant tissue-specific identifiers, it may be that whole-genome bisulfite sequencing (WGBS) provides an alternative to WGS for tissue deconvolution purposes. The complex array of data that can be obtained from diverse WGS analyses will represent multi-dimensional data to be subjected to feature extraction and various machine learning approaches. Ultimately, it will be possible to develop a model capable of distinguishing cfDNA that was derived from blood of a healthy individual and cfDNA derived from a patient with cancer. In the latter case, appropriate models may allow for tumor classification/subtyping, assessment of tumor evolution and identification of druggable targets or resistance markers. Furthermore, this would also open up exciting avenues for the analysis of cfDNA that extend beyond application in oncology

From these recent developments, we dare to provide a personal view as to how cfDNA will be analyzed in the future (Fig. 6). We envision that instead of panels, only WGS will be conducted with a moderate sequencing depth of 30-35x. Such a sequencing depth, using appropriate bioinformatics tools, will be sufficient to track hundreds to thousands of somatic tumor-associated mutations and copy number alterations in a personalized setting [48]. Fragmentation patterns, i.e., variability of cfDNA lengths, can be investigated for further evidence for the presence of ctDNA. From nucleosome position mapping, essential biological information can be inferred, such as which genes are expressed or which transcription factors are active in the tumor cells. These features will be fed into multi-parameter classification models to enable cfDNA analyses with unprecedented resolution. With the wealth of information that can be extracted from cfDNA WGS at 30-35x coverage, this cfDNA analysis strategy will be applicable to both early-stage and advanced-stage cancer diseases. Importantly, such cfDNA evaluation strategies may well extend beyond applications in cancer to a more general use, for example, for evaluating chronic diseases or the health status of an individual.

### Concluding remarks

Owing to its diverse non-invasive application, analysis of ctDNA represents a future standard practice of clinical oncology. Herein, we hope to have summarized the most current applications, particularly distinguishing between established use cases and those with promising future potential, in turn supporting clinicians who may order and rely on these molecular testing modalities to guide their patient care in real-time (for a summary of open issues and potential solutions, see the summary box in the Appendix). However, as the number and types of liquid biopsy testing grow, so, too, does the requirement for information across multiple, complex knowledge domains in order to understand these novel approaches. Although liquid biopsy is just one tool in the precision oncology arsenal, the general growing and overwhelming amount of “omics” data coupled with novel therapy indications has rendered critical point-of-care decisions for oncologists and other precision medicine players challenging. Such complexity creates a disconnect between the researchers who develop assays and associated bioinformatics analyses, the clinical practitioners who must keep up to date with validated testing strategies, payer institutions who must determine which tests represent medical necessity and thus justify reimbursement, and patients and their families who may struggle to understand how treatment decisions are made throughout their journey with cancer. In this regard, interdisciplinary solutions must be established to facilitate ongoing exchange between the various experts of precision medicine. Molecular tumor boards represent critical instruments for bringing together these decoupled knowledge domains to inform clinical decisions that ultimately benefit the patient and relieve healthcare systems [33, 67, 171–173]. Interestingly, although its importance is frequently emphasized at conferences and in other media [52, 174, 175], further education platforms for clinicians without access to MTBs are not prevalent, although young oncologists in particular would benefit immensely from learning about the fundamentals of genomics and liquid biopsy profiling early on in their training. We have recently begun one such initiative to generate high-quality content tailored to clinicians (https://www.vesseldna.com/a-clinicians-handbook-for-ctdna/). Similarly, most current oncology workflows overlook direct incorporation of the patient into critical decision-making processes, as most patients do not possess the necessary capacity to understand such decisions based on genomics data. It would be worthwhile to evaluate workflows geared towards patient education and personalized consultation such that the patient may better understand the interpretation of his or her molecular testing and liquid biopsy results which guide treatment decisions and potentially influence his or her clinical outcome. As the field of precision oncology and liquid biopsy methodology begin to mature, researchers, clinicians and patients may look forward to innovative opportunities in personalized cancer care.

## Data Availability

Not applicable.

## Summary box

### Why is analysis of ctDNA not yet a clinical gold standard? Open issues and potential solutions

**Table:**

**Type of issue**	**Bottleneck**	**Potential workaround/solution**
Logistical issues	Many hospitals face reimbursement issues when it comes to liquid biopsy testing and costs may be difficult to justify and thus are not covered for every ordered test.	This will hopefully change when significant progress is made in the analytical and clinical validity as well as clinical utility of plasma-based assays, especially as evidence from appropriately designed clinical trials accumulates. To date, there are no cost-effectiveness data on cfDNA assays and models for evaluating the economic impact on health must be considered in order for healthcare payers to cover such liquid biopsy solutions [176]
	Access to testing	Those who face reimbursement issues from large, commercial providers may find a solution in partnering with academic labs who perform validated assays routinely
Pre-analytical/analytical factors	Despite advances in liquid biopsy technology, pre-analytical and analytical standards are still lacking and may vary between testing labs. The 2018 joint review by ASCO and the College of American Pathologists determined that data on pre-analytic variables, analytical validity, interpretation and reporting are still insufficient to justify widespread adoption for the majority of ctDNA solid tumor assays [96].	Previous works [60, 62, 128, 177–182] have detailed the influence of pre-analytical factors such as blood collection tubes, plasma versus serum, transit time, centrifugation protocols, storage conditions, cfDNA purification, quantification, characterization and analysis platforms. Several collaborative efforts are currently underway to evaluate these findings, devise the criteria for standardizing liquid biopsy workflows, put these into context for providers and end users of cfDNA assays, as well as to bridge the gap between academia and industry to enable a transition of research findings into clinical implementation: The European Liquid Biopsy Society (ELBS); European Liquid Biopsy Academy (ELBA); International Society of Liquid Biopsy (ISLB); BloodPac
Negative results	According to the ASCO Post, the 2021 National Comprehensive Cancer Network (NCCN) Clinical Practice Guidelines in Oncology (NCCN Guidelines®) for molecular and biomarker analysis of NSCLC advises that cfDNA/ctDNA testing should not replace standard histologic diagnosis via tissue testing, unless the patient is unable to undergo invasive tissue sampling [183].	A recent consensus statement released by the International Association for the Study of Lung Cancer (IASLC) concluded that ctDNA analysis is an acceptable initial approach detection of biomarkers at diagnosis as well as for monitoring targeted therapies [184].
	Both technical and biological factors can influence the concordance between tumor tissue results and plasma DNA analysis.	The risk of false-positive and false-negative results from liquid biopsy testing have not completely eradicated the necessity of reflex tissue testing, such that the FDA recommends follow-up testing after receiving negative results, including those from commercial providers such as Guardant360 CDx and FoundationOne Liquid CDx
Diversity of NGS panels	Generally, there are no harmonized minimal requirements for NGS panels, although most harbor biomarkers within guidelines of national consortia, e.g. NCCN.	Implementation of broader NGS panels, e.g. those that evaluate mutations outside of the canonical hotspots, as these may reflect emerging biomarkers or inclusion criteria for recruiting/ongoing clinical trials
Clinical validity	Determination of clinical validity for broad NGS panels is challenging, as they are applied in multiple tumor types. For both of the use cases of therapy monitoring and early-stage cancer, evidence of clinical validity is still emerging, whereas there is still no evidence available for cancer screening purposes.	As summarized by Ignatiadis et al., further clinical validation of ctDNA assays will be conducted in the form of large-cohort phase III trials, which should include mandatory blood sampling and emphasize the need to report the results of liquid biopsy assays as official trial results [52].
Clinical utility	Currently, liquid biopsy assays lack consistency and precision. In fact, clinical validity and clinical utility have not yet been shown for the vast majority of assays.	Sophisticated multicenter clinical validation studies and regulatory guidelines are lacking but must be established to ensure responsible future application of liquid biopsies in precision oncology.
	It is not yet known whether interventional treatment based on detection of ctDNA relapse via liquid biopsy will actually improve patient outcome, i.e. cure, or whether systemic treatment will simply delay onset of metastatic disease [52].	Design and conducting of suitable clinical trials with the goal of improving outcomes of patients with detectable ctDNA
Bioinformatics	Increasing complexity of bioinformatics algorithms	Need for increased usability, e.g. through user-friendly graphical user interfaces with clear and concise instruction for use and interpretation of results
Education	Lack of structured, formal training available for clinicians regarding the implementation of genomic medicine: which tests to order for which patients; understanding the benefits and limitations of NGS assays; when to order liquid biopsy; how to interpret the clinical reports, prioritize variants and evidence and convert potential actionable insight into clinical care.	Such questions may be answered within the context of an appropriately set up molecular tumor board (MTB), where a panel of diverse experts, e.g. oncologists, pathologists, clinical geneticists and bioinformaticians meet on an ongoing basis to deliver the most suitable precision oncology approaches. Additionally, innovative education platforms, particularly geared towards young oncologists, may provide the fundamental awareness of such testing modalities early on in a clinician’s training. Molecular profiling and liquid biopsy education should be integrated into university training programs.
Interpretation bottlenecks	Lack of MTB infrastructure within hospital	With the advent of virtual molecular tumor boards, it may be possible to partner with experienced MTBs to discuss difficult patient cases
	Clinical decision support tools are still in their infancy and harmonization efforts regarding the interpretation of genomic variants have only just begun [124–126, 185] .	Various strategies of decision support software may lead to discrepancies in pathogenicity, actionability and treatment matching when interpreting genomic variants. For this reason, data must be evaluated and prioritized at a multidisciplinary MTB to derive the most suitable treatment decision.
	The interpretation and prioritization of variants at an MTB may vary from clinic to clinic and some MTBs may still be unfamiliar with data derived from ctDNA assays.	Clinics should strictly employ standardized criteria to define actionability and must rely on validated evidence-based scales, such as the FDA-approved content of OncoKB and European ESCAT guidelines [186–188]

## Abbreviations

ASCO: American Society of Clinical Oncology
CAPP-Seq: CAncer Personalized Profiling by deep Sequencing
cfDNA: Cell-free DNA
CGP: Comprehensive genomic profiling
CHIP: Clonal hematopoiesis of indeterminate potential
CRPC: Castration-resistant prostate cancer
ctDNA: Circulating tumor DNA
DHS: DNase hypersensitivity site
DNA: Deoxyribonucleic acid
dPCR: Digital PCR
ER: Estrogen receptor
FDA: Food and Drug Administration
HR: Hormone receptor
HRR: Homologous recombination repair
ICI: Immune checkpoint inhibitor
LOD: Limit of detection
mBC: Metastatic breast cancer
MCED: Multi-cancer early detection
mCRPC: Metastatic castration-resistant prostate cancer
MRD: Minimal residual disease
MTB: Molecular tumor board
NCCN: National Comprehensive Cancer Network
NDR: Nucleosome-depleted region
NGS: Next-generation sequencing
NSCLC: Non-small cell lung cancer
PARPi: Poly adenosine diphosphate-ribose polymerase inhibitor
RNA: Ribonucleic acid
ROI: Region of interest
Safe-SeqS: Safe Sequencing System
SBS: Sequencing-by-synthesis
SCNA: Somatic copy number alteration
SiMSen-Seq: Simple, multiplexed, PCR-based barcoding of DNA for sensitive mutation detection using sequencing
sWGS: Shallow whole-genome sequencing
TAm-Seq: Tagged Amplicon Sequencing
TARDIS: Targeted Digital Sequencing
TFBS: Transcription factor binding site
TKI: Tyrosine kinase inhibitor.
TSS: Transcription start site
VAF: Variant allele frequency
WGS: Whole-genome sequencing
